# Supplementary material for: Nongenetic surface engineering of mesenchymal stromal cells with polyvalent antibodies to enhance targeting efficiency
Source: Nat Commun. 2023 Sep 19;14:5806. doi: 10.1038/s41467-023-41609-8 (PMC10509227; doi:10.1038/s41467-023-41609-8)
Supplement: Supplementary file 1 — Supplementary Information [file 41467_2023_41609_MOESM1_ESM.pdf]

Supporting Information

Nongenetic surface engineering of mesenchymal stromal cells with polyvalent antibodies to enhance targeting efficiency

Supplementary Tables

Supplementary Table 1. Quantitation of DI on K562 cells surface.

Supplementary Table 2. Quantitation of DI on MSCs surface.

Supplementary Table 3. The comparison of PAV-engineered MSCs with previously reported engineered MSCs for the treatment of mice with colitis.

Supplementary Table 4. The procedures and timeline for the preparation of PAV-engineered cells.

Supplementary Table 5. The cost of preparing one million PAV-engineered cells.

Supplementary Table 6. DNA sequences.

Supplementary Figures

Supplementary Figure 1. Schematic illustration of sequences and secondary structure of DI and DNA monomers.

Supplementary Figure 2. DNA sequence screening.

Supplementary Figure 3. Effect of incubation time on antibody assembly.

Supplementary Figure 4. The stability analysis of the surface modifications.

Supplementary Figure 5. Adhesion of engineered K562 cells to C166 cells under static conditions.

Supplementary Figure 6. Adhesion of engineered MSCs to C166 cells under flow condition.

Supplementary Figure 7. Shedding of antibodies after MSC migration.

Supplementary Figure 8. Effect of surface engineering on MSCs proliferation and adhesion.

Supplementary Figure 9. Biosafety analysis of engineered MSCs.

Supplementary Figure 10. Toxicity analysis.

Supplementary Figure 11. Immunofluorescence staining of the ear tissues.

Supplementary Figure 12. Quantitation of fluorescence intensity in inflammatory ear via IVIS imaging.

Supplementary Figure13. Confocal fluorescence imaging of the mouse ear.

Supplementary Figure 14. Biodistribution of engineered MSCs in mouse model of acute ear inflammation.

Supplementary Figure 15. Immunofluorescence staining of colon tissue.

Supplementary Figure 16. Biodistribution of engineered MSCs in mice with colitis.

DI concentration (μM)	DI density (units per cell)
0.05	2.5×10^6
0.075	4.7×10^6
0.1	5.5×10^6
0.25	8.3×10^6
0.5	1.6×10^7
1	3.3×10^7
2	4.3×10^7

Supplementary Table 1. Quantitation of DI on K562 cells surface.

DI concentration (μM)	DI density (units per cell)
0.05	1.4×10^6
0.075	2.0×10^6
0.1	3.3×10^6
0.25	6.2×10^6
0.5	1.3×10^7
1	2.7×10^7
2	3.5×10^7

Supplementary Table 2. Quantitation of DI on MSCs surface.

References	Targeting efficiency	Therapeutic effects					
		Body weight	DAI	Colon length in colitis mice vs. normal mice	MPO activity in colitis mice vs. normal mice	Levels of TNF-α，IL-6，IL-10 in colitis mice vs. normal mice	Histological sections or pathological scores
PAV engineered-MSCs for colitis	4.67%	103%	1	0.92	1.1	1;0.91;0.93	virtually normal pathological structures
Anti-VCAM1-coated MSCs for colitis[1]	NA	~95%	NA	~0.8	NA	NA	pathological scores 2.5
Anti-VCAM1-coated MSCs for colitis[2]	NA	<95%	~1	NA	NA	NA	few inflammatory response
IFN-γ expressing MSCs by genetic engineering for colitis[3]	NA	~80%	~1.5	NA	NA	NA	pathological scores >1.5
IL1β-pretreated MSCs for colitis[4]	NA	~95%	~2.5	~0.8	NA	NA	pathological scores 2
ICAM-1 overexpressing MSCs by genetic engineering for colitis[5]	NA	~90%	NA	~0.85	NA	NA	pathological scores >2
SDF-1-pretreated MSCs for colitis[6]	NA	~95%	~1.5	~0.85	NA	~1; NA; ~1.3	hyperemia and edema were remitted; inflammatory cell infiltration was decreased
IFN-γ+ IL1β-pretreated MSCs for colitis[7]	NA	~90%	NA	~0.85	NA	NA	structural disruption of crypt foci
TLR-3-pretreated MSCs for colitis for colitis[8]	NA	~85%	~5	~0.86	NA	NA	pathological scores >2
Roe-inspired MSC microcapsules for colitis[9]	NA	~95%	~2	~0.93	~1.5	~4; ~1.5; ~0.75	pathological scores >3.5
ASA-pretreated MSCs for colitis[10]	NA	NA	~5	NA	NA	NA; NA; ~2.5	infiltration of inflammatory cells; pathological scores >4

Supplementary Table 3. The comparison of PAV-engineered MSCs with previously reported engineered MSCs for the treatment of mice with colitis.

Cell engineering procedure	Time
DI co-incubation with cells	20min
HCR	3h
Centrifuging, washing	<30min
All	<4h

Supplementary Table 4. The procedures and timeline for the preparation of PAV-engineered cells.

Major reagents	Costs ($)
DI-cholesterol (0.4 nmol)	0.6
DM1-antiVCAM1 (1.8 μg)	3
DM2-antiVCAM1 (1.8 μg)	3
Consumables	<0.5
All	8

Supplementary Table 5. The cost of preparing one million PAV-engineered cells.

DNA name	Sequence (5-->3)
**1-DI**	CCTCATCCCACTCCTACCTAAACCTTTTTTTTTTTTTTTT TTTT/3 cholesterol
**1-DM1**	GGTTTAGGTAGGAGTGGGATGAGGCCAAATCCTCATCC CACTCCTACC/ 3`NH_2 C6
**1-DM2**	CCTCATCCCACTCCTACCTAAACCGGTAGGAGTGGGAT GAGGATTTGG/ 5`NH_2 C6
**2-DI**	AGATCCAAATAGCACGCCTGAAGTTTTTTTTTTTTTTTT TTTT/3 cholesterol
**2-DM1**	AATAGCACGCCTGAAGATTGGAGCTTCAGGCGTGCTAT TTGGATCT/ 3`NH_2 C6
**2-DM2**	CTCCAATCTTCAGGCGTGCTATTAGATCCAAATAGCACG CCTGAAG/5`NH_2 C6
**3-DI**	CGTCGGAAACAAATGGCTAATCGTTTTTTTTTTTTTTTTT TTT/3 cholesterol
**3-DM1**	GGTAAACAAAGGCTGCTCCAGAAGCAGCCTTTGTTTAC CGATTAGC/3`NH_2 C6
**3-DM2**	TTCTGGAGCAGCCTTTGTTTACCGCTAATCGGTAAACA AAGGCTGC/5`NH_2 C6
**4-DI**	ATTCTCGAGGGCTAATGGATGTTTTTTTTTTTTTTTTTT TTT/3 cholesterol
**4-DM1**	TGGCGAGGGACTTTATAGGGTATATAAAGTCCCTCGCCA TCTCAGT/3`NH_2 C6
**4-DM2**	ATACCCTATAAAGTCCCTCGCCAACTGAGATGGCGAGG GACTTTAT/5`NH_2 C6
**5-DM1**	GGTTTAGGTAGGAGTGGGATGAGGCCAAATCCTCATCC CACTCCTACCATTTT/ 3`NH_2 C6
**5-DM2**	TTTTACCTCATCCCACTCCTACCTAAACCGGTAGGAGTG GGATGAGGATTTGG/ 5`NH_2 C6

Supplementary Table 6. DNA sequences.

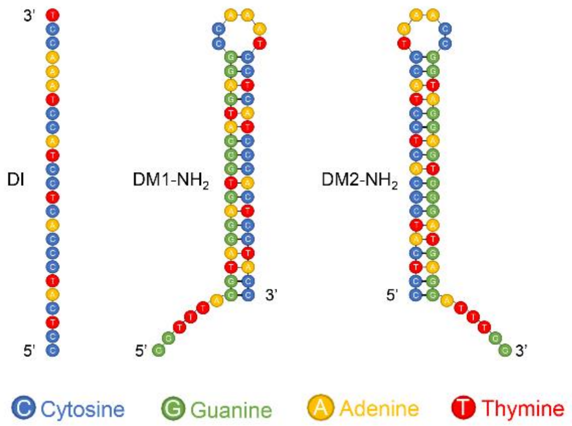

Supplementary Figure 1. Schematic illustration of sequences and secondary structure of DI and DNA monomers.

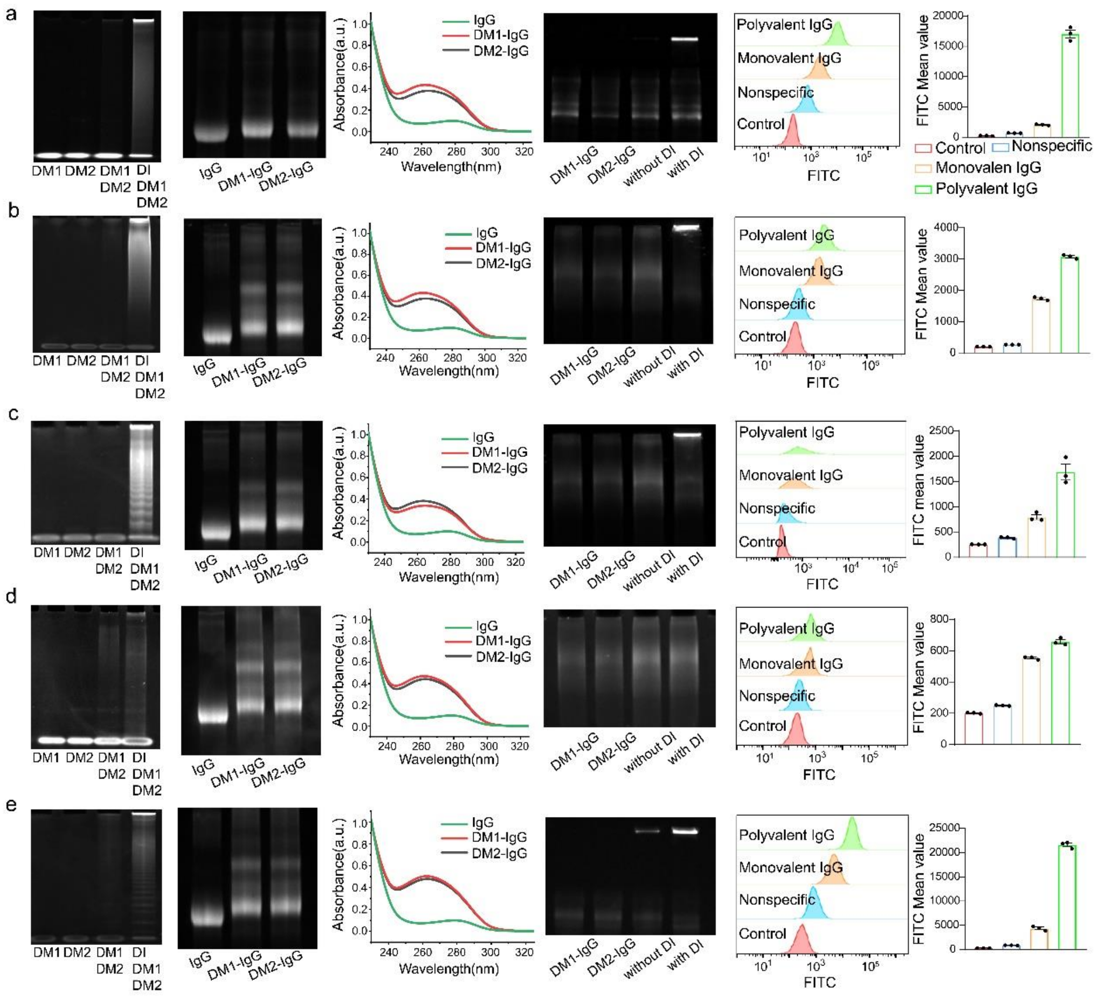

Supplementary Figure 2. DNA sequence screening. Five sets of DNA sequences were designed to program antibody assembly via HCR (a-e). From left to right: electrophoresis gel image of DNA polymers; electrophoresis gel image of DNA-IgG; UV-vis absorption spectra of DNA-IgG; electrophoresis gel image of polyvalent IgG; Flow cytometry analysis of the fluorescence intensity of K562 cells modified with monovalent or polyvalent IgG; quantitation of fluorescence intensity. Mean ± SEM, n=3 independent replicates. Source data are provided as a Source Data file.

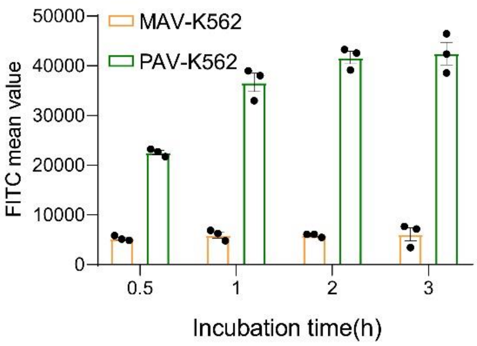

Supplementary Figure 3. Effect of incubation time on antibody assembly. Mean ± SEM, n=3 independent experiments. Source data are provided as a Source Data file.

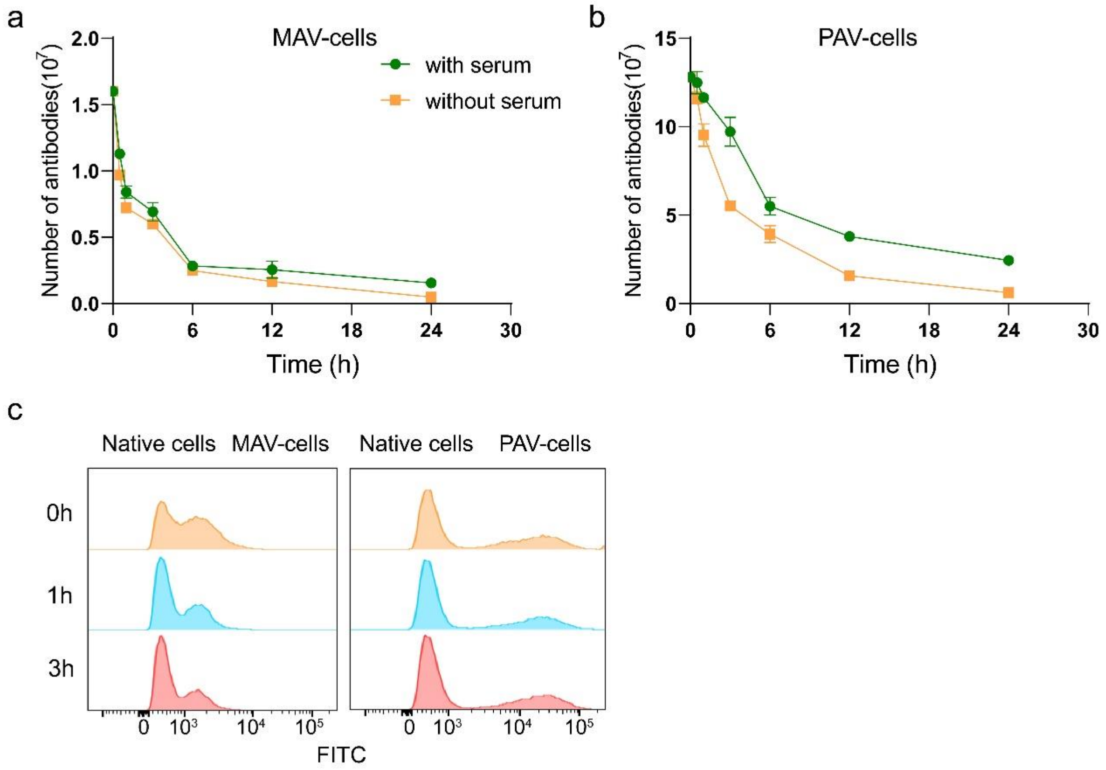

Supplementary Figure 4. The stability analysis of the surface modifications. a and b) Residual levels of antibodies in MAV- or PAV-cells cultured in serum or serum-free medium. The cells were modified at a density of 1.6×10^{7} DI units/cell. Following monovalent or polyvalent engineering, the cells were cultured in medium supplemented with either 10% serum or no serum. Residual fluorescence intensity on the cell surface was detected by flow cytometry at corresponding time points. Mean ± SEM, n= 3 independent experiments. c) Flow cytometric analysis of the fluorescence intensity of native cells following 3 h of co-culture with MAV- or PAV-cells. n= 3 independent experiments. Source data are provided as a Source Data file.

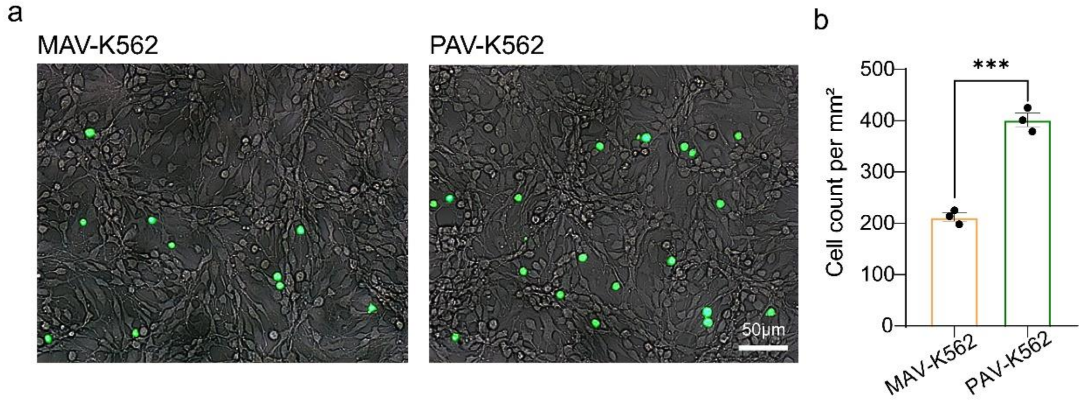

Supplementary Figure 5. Adhesion of engineered K562 cells to C166 cells under static conditions. a) Representative images showing MAV- or PAV-engineered K562 cells adhering to C166 cells. Representative images out of 7 images obtained are shown. b) Quantitative analysis of the numbers of adherent cells in MAV and PAV group. ***: P =0.0003. Mean ± SEM, n= 3 independent experiments. Statistical analysis was performed by two-tailed unpaired t-test (**P<0.01; ***P<0.001; ****P<0.0001; NS, non-significant). Source data are provided as a Source Data file.

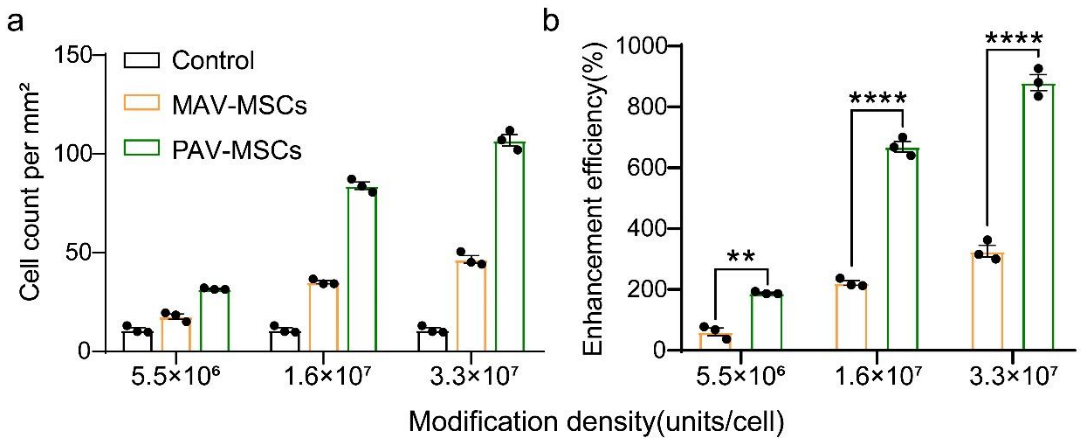

Supplementary Figure 6. Adhesion of engineered MSCs to C166 cells under flow condition. a) Number of engineered MSCs adhering to C166 cells. MSCs were modified with different density of MAV or PAV on the cell surface and tested at the same shear stress of 4 dyn/cm^2. b) Quantitation of the enhanced efficiency in MAV and PAV group. **: P=0.0013, ****: P<0.0001. For a) and b), Mean ± SEM, n=3 independent replicates. Statistical analysis was performed by one-way ANOVA with Tukey's multiple comparisons tests (**P<0.01; ***P<0.001; ****P<0.0001; NS, nonsignificant). Source data are provided as a Source Data file.

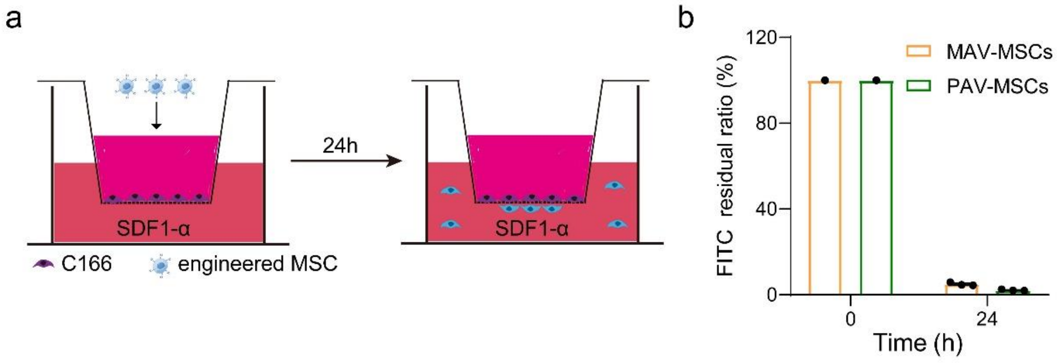

Supplementary Figure 7. Shedding of antibodies after MSC migration. a) Schematic illustration of migration assay in transwell. b) Percentage of residual fluorescence intensity on engineered MSCs pre- (0 h) and post-migration (24 h). Mean ± SEM, n=3 independent replicates. Source data are provided as a Source Data file.

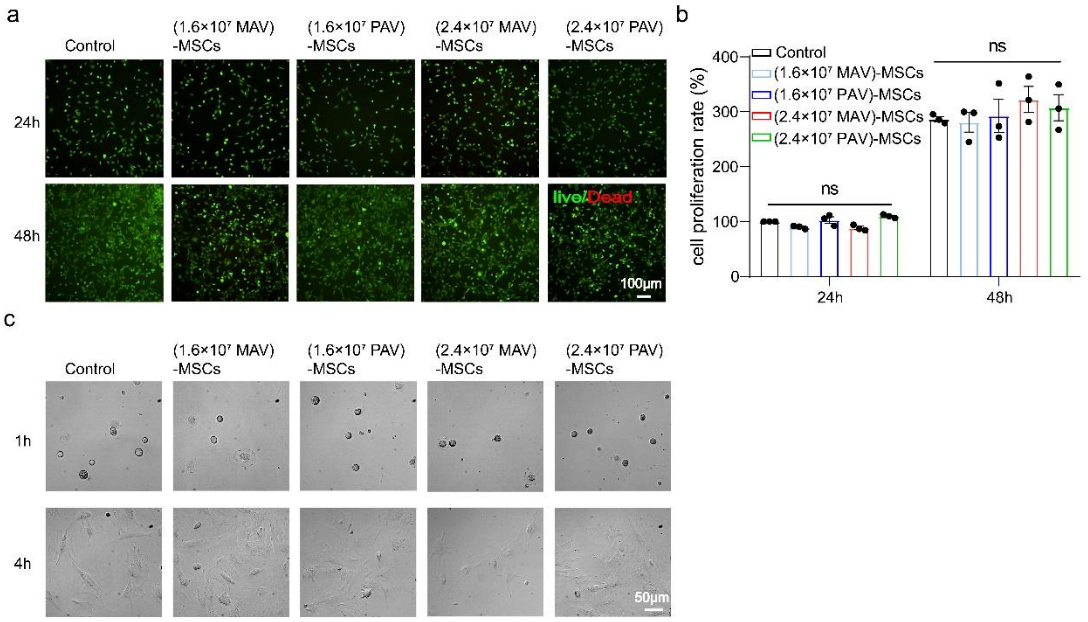

Supplementary Figure 8. Effect of surface engineering on MSCs proliferation and adhesion. a) Representative Live/Dead staining images of native MSCs and engineered MSCs. b) CCK-8 assays for analyzing the proliferation of native and engineered MSCs. Mean ± SEM, n=3 independent experiments. c) Representative images of cell adhesion. The phase-contrast microscopy showed that there were no differences in cell morphology between unmodified and engineered MSCs after 1 or 4 hours of incubation. For a) and c), representative images out of 7 images obtained are shown. n=3 biological replicates. Statistical analysis was performed by two-way ANOVA with Tukey's multiple comparisons tests (***P*<0.01; ****P*<0.001; *****P*<0.0001; NS, nonsignificant). Source data are provided as a Source Data file.

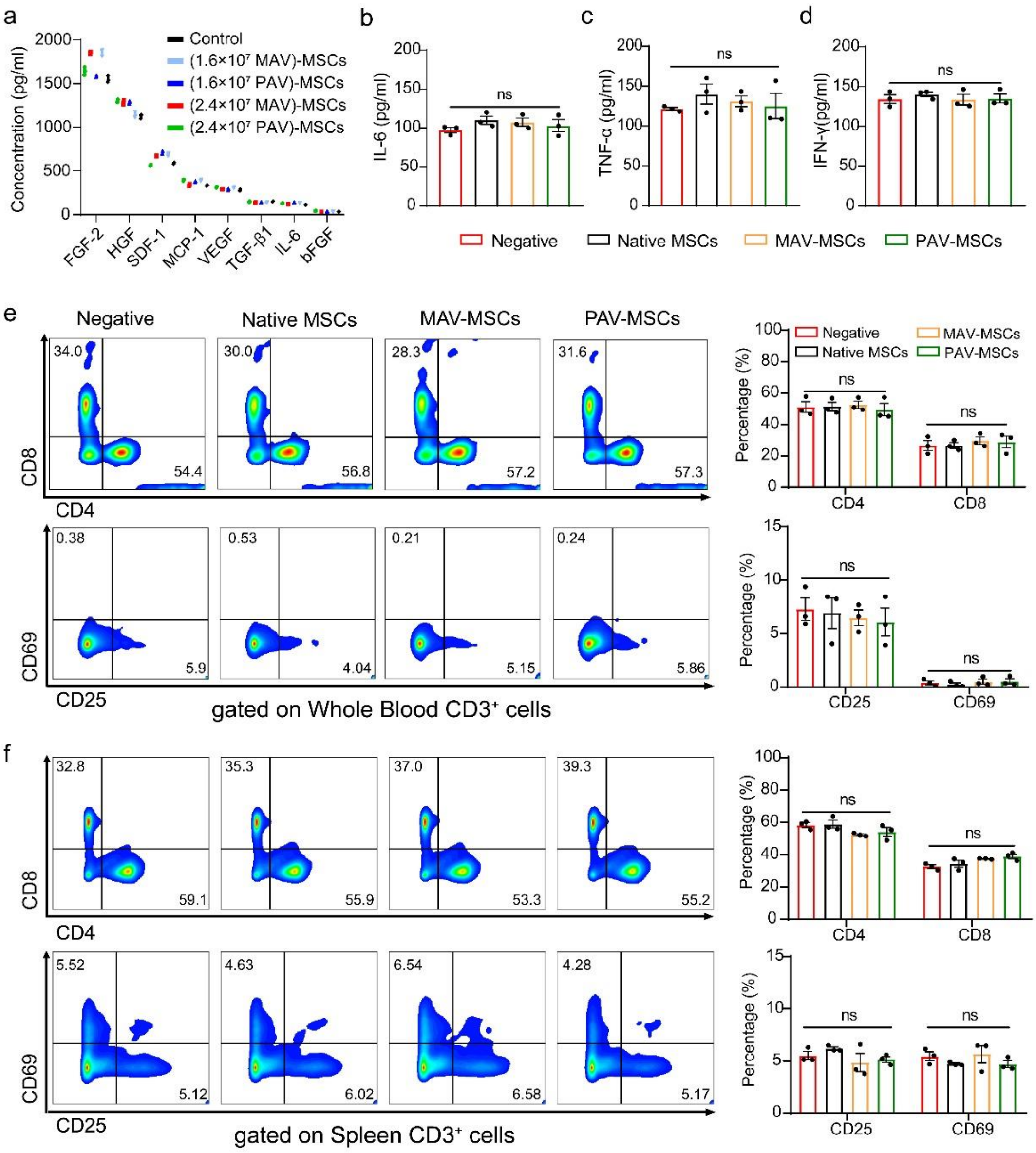

Supplementary Figure 9. Biosafety analysis of engineered MSCs. a) Cytokine secretion profiles of engineered MSCs. Cells at Passage 3 were grown in basal media for 4 days, and secreted proteins were assessed by ELISA. Mean ± SEM, n=3 independent experiments. b-d) Serum levels of interleukin-6 (IL-6), tumor necrosis factor alpha (TNF-α) and interferon gamma (IFN-γ) 48 h after the administration of native or engineered MSCs to healthy mice. Mean ± SEM, n=3 mice. e and f) Flow data (left) and quantitative analysis (right) of the proportion of T cells expressing CD4, CD8, CD25, and CD69. Healthy BALB/c mice were intravenously injected with native MSCs or engineered MSCs. After 48 h, the mice were sacrificed, and whole blood (e) and spleen tissue (f) were collected for flow cytometric analysis. Mean ± SEM, n=3 mice.

Statistical analysis was performed by one-way ANOVA with Tukey's multiple comparisons tests (***P*<0.01; ****P*<0.001; *****P*<0.0001; NS, nonsignificant). Source data are provided as a Source Data file.

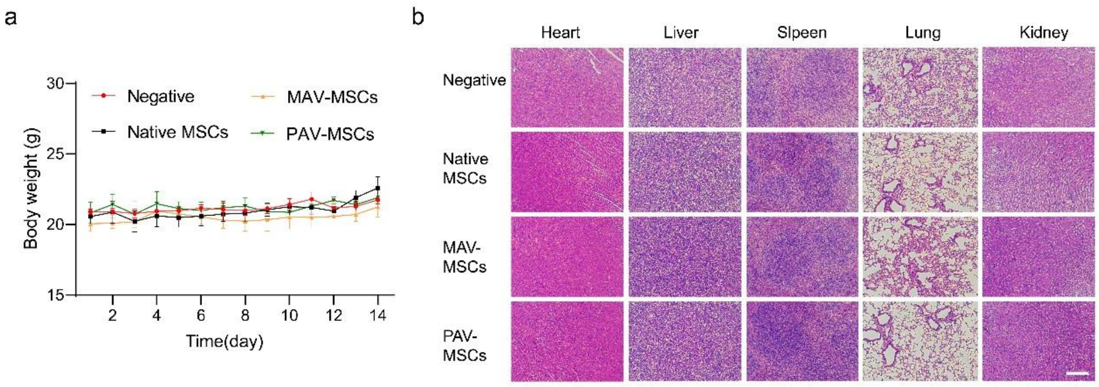

Supplementary Figure 10. Toxicity analysis. a) Body weight change of the mice receiving different treatments. b) Representative H&E images of different organs collected from mice of indicated groups. The healthy mice were randomly divided into four groups: Negative, Native MSCs, MAV-MSCs and PAV-MSCs. Body weight of mice were measured after intravenous administration of MSCs. After 14 days, the mice were sacrificed to remove the major organs for H&E staining. Mean ± SEM, n=3 mice. Scale bar: 200 µm. Source data are provided as a Source Data file.

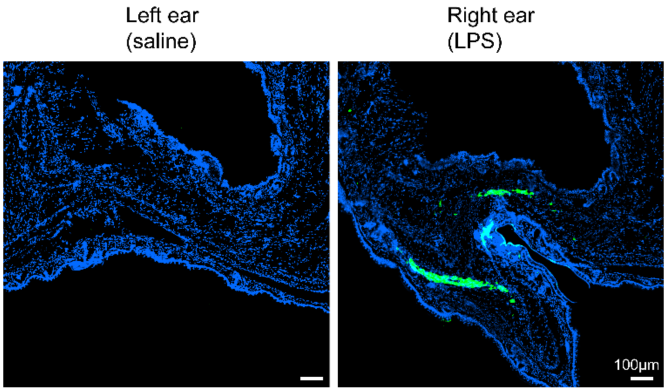

Supplementary Figure 11. Immunofluorescence staining of the ear tissues. Mice were intradermally injected with LPS into the right ear to induce acute inflammation. Mouse ears were collected and whole-mount stained with anti-VCAM1 (green) and DAPI (blue). Representative images out of 7 images obtained are shown.

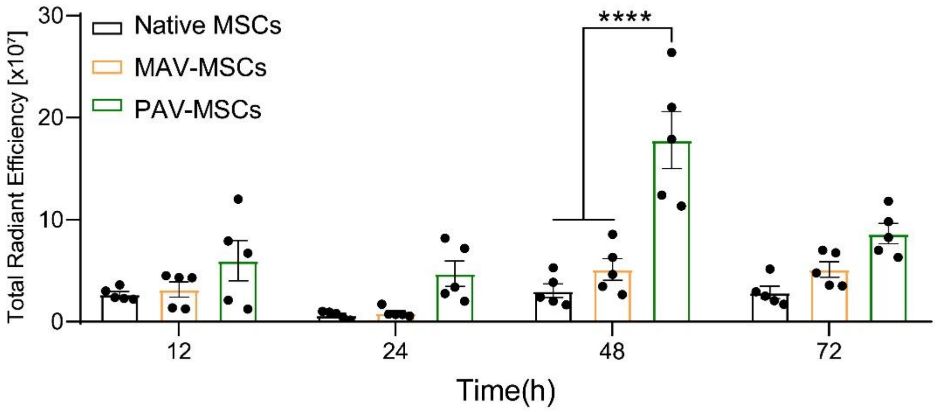

Supplementary Figure 12. Quantitation of fluorescence intensity in inflammatory ear via IVIS imaging. ****: *P*<0.0001. Mean ± SEM, n=5 mice. Statistical analysis was performed by one-way ANOVA with Tukey's multiple comparisons tests (***P*<0.01; ****P*<0.001; *****P*<0.0001; NS, nonsignificant). Source data are provided as a Source Data file.

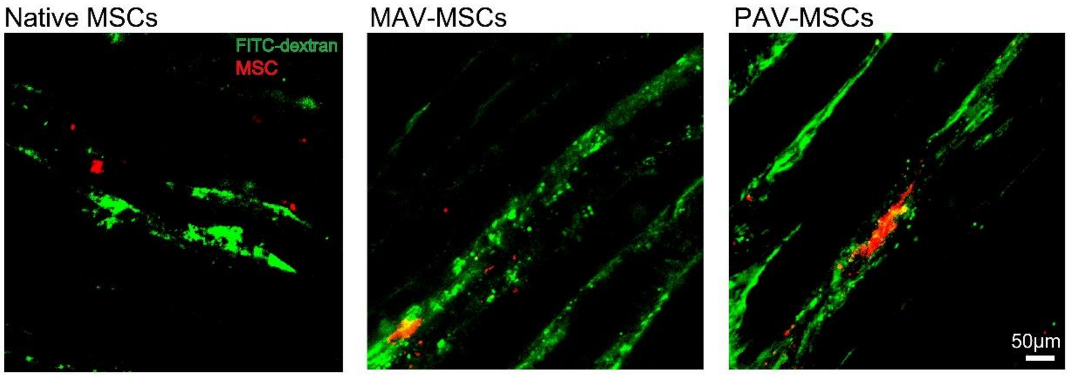

Supplementary Figure 13. Confocal fluorescence imaging of the mouse ear. Mice were i.v. injected with 1×10^6 native or engineered MSCs. Then confocal fluorescence images of the right ear of mice were obtained at 48h. FITC-dextran was injected intravenously 2h before imaging. Red: MSCs, Green: Blood vessels. Representative images out of 7 images obtained are shown.

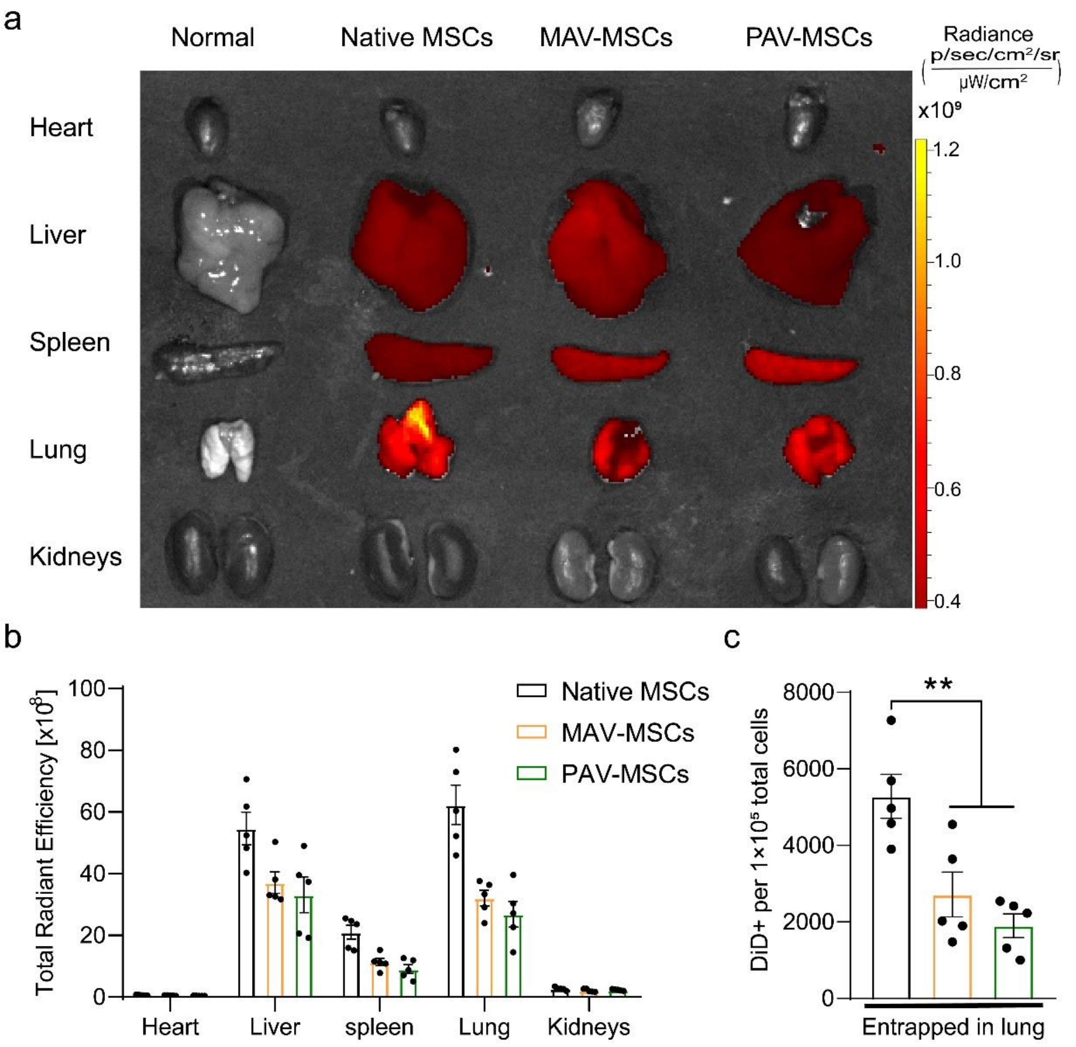

Supplementary Figure 14. Biodistribution of engineered MSCs in mouse model of acute ear inflammation. a) IVIS imaging of the mouse organs. b) Quantitation of fluorescence intensity of organs in different groups. Mice were euthanized at 48 h after intravenous injection of 1×10^6 DiD-labelled native or engineered MSCs and the organs were removed for fluorescence imaging with IVIS Lumina Series III. Mean ± SEM. c) Number of DiD+ MSCs out of 1×10^5 total cells collected from mouse lungs at 48 h after administration. **: P=0.0014. Mean ± SEM. For a), b) and c), n=5 mice. Statistical analysis was performed by one-way ANOVA with Tukey's multiple comparisons tests (**P<0.01; ***P<0.001; ****P<0.0001; NS, nonsignificant). Source data are provided as a Source Data file.

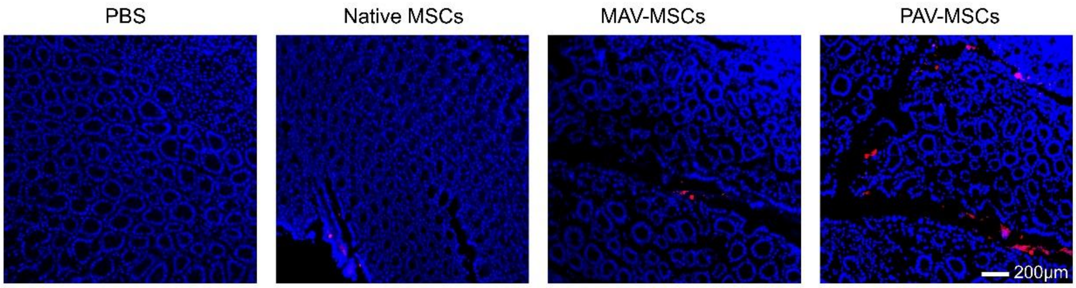

Supplementary Figure 15. Immunofluorescence staining of colon tissue. Immunofluorescence staining of the colon at 48h post administration for analyzing the MSCs homing. Red: MSCs, Blue: DAPI. Representative images out of 7 images obtained are shown.

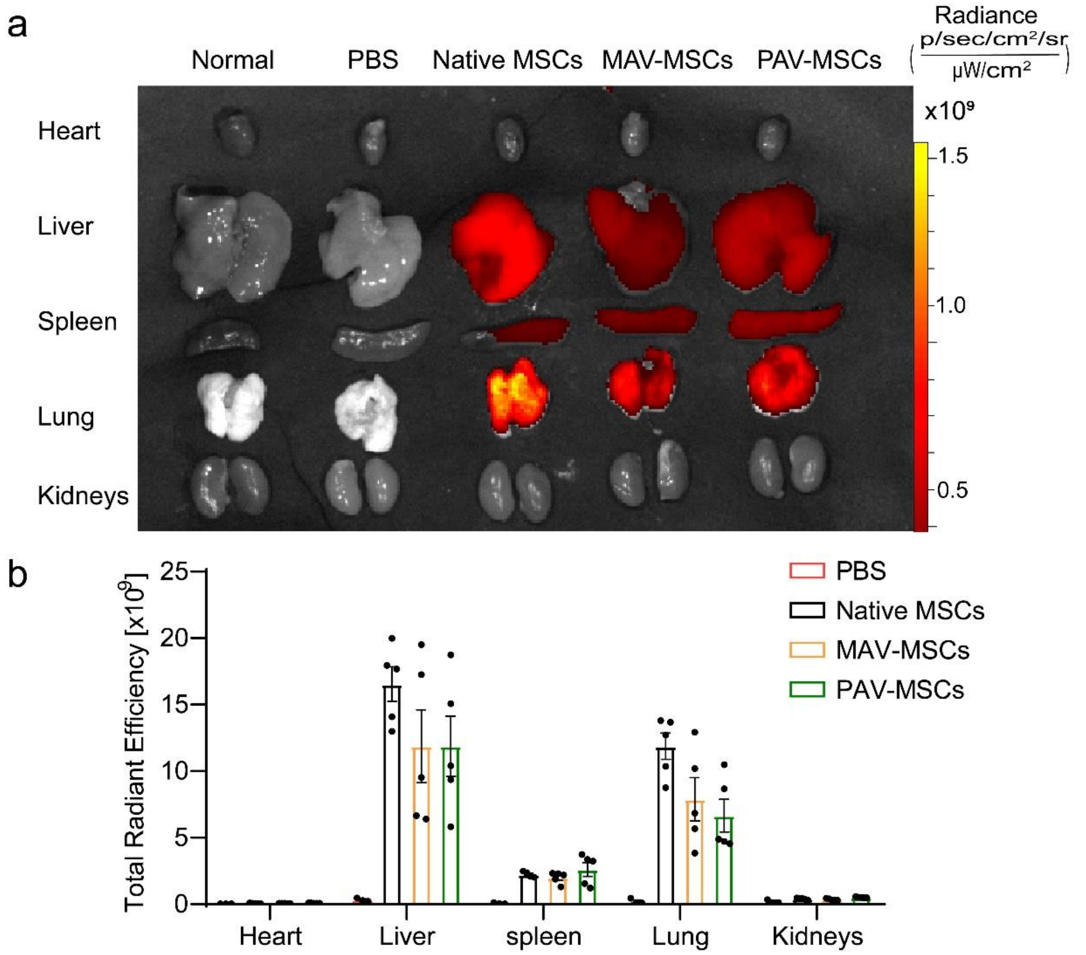

Supplementary Figure 16. Biodistribution of engineered MSCs in mice with colitis. a) IVIS imaging of the mouse organs. b) Quantitation of fluorescence intensity of organs in different groups. Mice were euthanized at 48 h after intravenous injection of 1×10^6 DiD-labelled native or engineered MSCs and the organs were removed for fluorescence imaging with IVIS Lumina Series III. Mean ± SEM, n=5 mice. Source data are provided as a Source Data file.

Supplementary References

1. Ko IK, et al. Targeting improves MSC treatment of inflammatory bowel disease. *Mol Ther* **18**, 1365-1372 (2010).
2. Chen Q., Li Y., Chen Z., Du H., Wan J. Anti-VCAM 1 Antibody-Coated Mesenchymal Stromal Cells Attenuate Experimental Colitis via Immunomodulation. *Med Sci Monit* **25**, 4457-4468 (2019).
3. Chen Y. et al. Gene delivery with IFN-γ-expression plasmids enhances the therapeutic effects of MSCs on DSS-induced mouse colitis. *Inflamm Res* **64**, 671-681 (2015).
4. Fan H. et al. Pre-treatment with IL-1β enhances the efficacy of MSC transplantation in DSS-induced colitis. *Cell Mol Immunol* **9**, 473-481 (2012).
5. Li X. et al. Intercellular adhesion molecule-1 enhances the therapeutic effects of MSCs in a dextran sulfate sodium-induced colitis models by promoting MSCs homing to murine colons and spleens. *Stem Cell Res Ther* **10**, 267 (2019).
6. Li X. et al. SDF-1/CXCR4 axis enhances the immunomodulation of human endometrial regenerative cells in alleviating experimental colitis. *Stem Cell Res Ther* **10**, 204 (2019).
7. Yu Y. et al. Preconditioning with interleukin-1β and interferon-γ enhances the efficacy of human umbilical cord blood-derived mesenchymal stem cells-based therapy via enhancing prostaglandin E2 secretion and indoleamine 2,3-dioxygenase activity in dextran sulfate sodium-induced colitis. *J Tissue Eng Regen Med* **13**, 1792-1804 (2019).
8. Fuenzalida P. et al. Toll-like receptor 3 pre-conditioning increases the therapeutic efficacy of umbilical cord mesenchymal stromal cells in a dextran sulfate sodium-induced colitis model. *Cytotherapy* **18**, 630-641 (2016).
9. Chen G., Wang F., Nie M., Zhang H., Zhang H., Zhao Y. Roe-inspired stem cell microcapsules for inflammatory bowel disease treatment. *Proc. Natl. Acad Sci. U S A* **118**, e2112704118 (2021).
10. Tang J. et al. Aspirin treatment improved mesenchymal stem cell immunomodulatory properties via the 15d-PGJ2/PPARγ/TGF-β1 pathway. *Stem Cells Dev* **23**, 2093-2103 (2014).
