# Supplementary material for: Nongenetic surface engineering of mesenchymal stromal cells with polyvalent antibodies to enhance targeting efficiency
Source: Nat Commun. 2023 Sep 19;14:5806. doi: 10.1038/s41467-023-41609-8 (PMC10509227; doi:10.1038/s41467-023-41609-8)
Supplement: Supplementary file 3 — Reporting Summary [file 41467_2023_41609_MOESM3_ESM.pdf]

nature portfolio

Corresponding author(s): Peng Shi

Last updated by author(s): Aug 28, 2023

Reporting Summary

Nature Portfolio wishes to improve the reproducibility of the work that we publish. This form provides structure for consistency and transparency in reporting. For further information on Nature Portfolio policies, see our Editorial Policies and the Editorial Policy Checklist.

Statistics

For all statistical analyses, confirm that the following items are present in the figure legend, table legend, main text, or Methods section.

n/a	Confirmed	
☐	☒	The exact sample size (*n*) for each experimental group/condition, given as a discrete number and unit of measurement
☐	☒	A statement on whether measurements were taken from distinct samples or whether the same sample was measured repeatedly
☐	☒	The statistical test(s) used AND whether they are one- or two-sided<br>*Only common tests should be described solely by name; describe more complex techniques in the Methods section.*
☒	☐	A description of all covariates tested
☐	☒	A description of any assumptions or corrections, such as tests of normality and adjustment for multiple comparisons
☐	☒	A full description of the statistical parameters including central tendency (e.g. means) or other basic estimates (e.g. regression coefficient) AND variation (e.g. standard deviation) or associated estimates of uncertainty (e.g. confidence intervals)
☐	☒	For null hypothesis testing, the test statistic (e.g. *F*, *t*, *r*) with confidence intervals, effect sizes, degrees of freedom and *P* value noted<br>*Give P values as exact values whenever suitable.*
☒	☐	For Bayesian analysis, information on the choice of priors and Markov chain Monte Carlo settings
☒	☐	For hierarchical and complex designs, identification of the appropriate level for tests and full reporting of outcomes
☒	☐	Estimates of effect sizes (e.g. Cohen's *d*, Pearson's *r*), indicating how they were calculated

Our web collection on statistics for biologists contains articles on many of the points above.

Software and code

Policy information about availability of computer code

Data collection	All the software are commercially available, including IVIS Lumina Series III Living Image v4.3.1, BD FACSDiva Software, and Zen black of Zeiss.
Data analysis	IVIS Lumina Series III Living Image v4.3.1 was used for the analysis of animal fluorescence images. Flowjo V10.0.7 was used for flow cytometry data analysis. ZEN 2 (blue edition) was used for the analysis of images from CLSM.<br>The statistical analysis was performed using GraphPad Prism 8.

For manuscripts utilizing custom algorithms or software that are central to the research but not yet described in published literature, software must be made available to editors and reviewers. We strongly encourage code deposition in a community repository (e.g. GitHub). See the Nature Portfolio guidelines for submitting code & software for further information.

Data

Policy information about availability of data

All manuscripts must include a data availability statement. This statement should provide the following information, where applicable:

- Accession codes, unique identifiers, or web links for publicly available datasets
- A description of any restrictions on data availability
- For clinical datasets or third party data, please ensure that the statement adheres to our policy

The data that support this study are available within the Article, Supplementary Information or Source data files. Source data are provided with this paper.

Human research participants

Policy information about studies involving human research participants and Sex and Gender in Research.

Reporting on sex and gender	Not applicable
Population characteristics	Not applicable
Recruitment	Not applicable
Ethics oversight	Not applicable

Note that full information on the approval of the study protocol must also be provided in the manuscript.

Field-specific reporting

Please select the one below that is the best fit for your research. If you are not sure, read the appropriate sections before making your selection.

☒ Life sciences ☐ Behavioural & social sciences ☐ Ecological, evolutionary & environmental sciences

For a reference copy of the document with all sections, see nature.com/documents/nr-reporting-summary-flat.pdf

Life sciences study design

All studies must disclose on these points even when the disclosure is negative.

Sample size	No statistical method was used to predetermine sample size. All in vitro data were done at least in triplicate and SEM were reported as stated in the figure and figure legends. In vivo experiments, according to the replacement, reduction and refinement principles of animal ethics regulation, the sample size was set to ensure the tests' reproducibility. Sample size was estimated on the basis of similar research reported in the literature:<br>Zhou, J., Li, M., Chen, Q. et al. Programmable probiotics modulate inflammation and gut microbiota for inflammatory bowel disease treatment after effective oral delivery. Nat Commun 13, 3432 (2022).
Data exclusions	On principle, data were only excluded for failed experiments.
Replication	Experimental findings were reliably reproduced. All experiments were performed at least in triplicates (see sample size of each experiment).
Randomization	All samples and animals were assigned randomly to experimental and control groups.
Blinding	No formal blinding was used in this study. In vitro experiments are usually performed by a single experimentalist. The principle of random assignment is used in in vivo experiments, colleagues aiding in data collection were blinded. The data analyses were based on objectively measurable data.

Reporting for specific materials, systems and methods

We require information from authors about some types of materials, experimental systems and methods used in many studies. Here, indicate whether each material, system or method listed is relevant to your study. If you are not sure if a list item applies to your research, read the appropriate section before selecting a response.

Materials & experimental systems

n/a	Involved in the study	
☐	☒	Antibodies
☐	☒	Eukaryotic cell lines
☒	☐	Palaeontology and archaeology
☐	☒	Animals and other organisms
☒	☐	Clinical data
☒	☐	Dual use research of concern

Methods

n/a	Involved in the study	
☒	☐	ChIP-seq
☐	☒	Flow cytometry
☒	☐	MRI-based neuroimaging

Antibodies

Antibodies used	FITC-labeled anti-mouse VCAM1 antibody (Biolegend, Catalog #: 105706);

Antibodies used	Ultra-LEAF™ Purified anti-mouse VCAM1 antibody (Biolegend, Catalog #: 105728);<br>FITC-labeled anti-mouse CD31 antibody (Biolegend, Catalog #: 102506);<br>FITC-labeled anti-mouse CD3 antibody (Biolegend, Catalog #: 100204);<br>Percp-labeled anti-mouse CD4 antibody (Biolegend, Catalog #: 100434);<br>Alexa Fluor® 700-labeled anti-mouse CD8 antibody (Biolegend, Catalog #: 100730);<br>APC-labeled anti-mouse CD69 antibody (Biolegend, Catalog #: 104514);<br>PE-labeled anti-mouse CD25 antibody (Biolegend, Catalog #: 101903)
Validation	The antibodies were validated by the manufacturers,all validation statements can be found on the respective antibody website:<br>FITC anti-mouse VCAM1 (Catalog #: 105706; Clone number: 429; Species Reactivity: mouse; Applications: Flow) : https://www.biolegend.com/en-us/products/fitc-anti-mouse-cd106-antibody-137;<br>Ultra-LEAF™ anti-mouse VCAM1 (Catalog #: 105728; Clone number: 429; Species Reactivity: mouse; Applications: Flow, IF, IP) : https://www.biolegend.com/en-us/products/ultra-leaf-purified-anti-mouse-cd106-antibody-18659;<br>FITC anti-mouse CD31 (Catalog #: 102506; Clone number: MEC13.3; Species Reactivity: mouse; Applications: Flow, IF, IP) : https://www.biolegend.com/en-us/products/fitc-anti-mouse-cd31-antibody-377;<br>FITC anti-mouse CD3 (Catalog #: 100204; Clone number: 17A2; Species Reactivity: mouse; Applications: Flow) : https://www.biolegend.com/en-us/products/fitc-anti-mouse-cd3-antibody-45;<br>Percp anti-mouse CD4 (Catalog #: 100434; Clone number: GK1.5; Species Reactivity: mouse; Applications: Flow) : https://www.biolegend.com/en-us/products/percp-cyanine5-5-anti-mouse-cd4-antibody-4220;<br>Alexa Fluor® 700 anti-mouse CD8 (Catalog #: 100730; Clone number: 53-6.7; Species Reactivity: mouse; Applications: Flow) : https://www.biolegend.com/en-us/products/alexa-fluor-700-anti-mouse-cd8a-antibody-3387;<br>APC anti-mouse CD69 (Catalog #: 104514; Clone number: H1.2F3; Species Reactivity: mouse; Applications: Flow, IP) : https://www.biolegend.com/en-us/products/apc-anti-mouse-cd69-antibody-3169;<br>PE anti-mouse CD25 (Catalog #: 101903; Clone number: 3C7; Species Reactivity: mouse; Applications: Flow) : https://www.biolegend.com/en-us/products/pe-anti-mouse-cd25-antibody-129.

Eukaryotic cell lines

Policy information about cell lines and Sex and Gender in Research

Cell line source(s)	Vascular endothelial cells C166 (ATCC, CRL-2581) and chronic myelogenous leukemia K-562 cells (ATCC, CCL-243) were obtained form ATCC. Mouse primary MSCs were purchased from iCell Bioscience Inc (MIC-iCell-s018).
Authentication	MSCs were authenticated by morphology and immunofluorescence staining. C166 and K562 cell lines were authenticated by STR profiling.
Mycoplasma contamination	All cell lines tested negative for mycoplasma contamination.
Commonly misidentified lines<br>(See ICLAC register)	No commonly misidentified cell lines were used in the study.

Animals and other research organisms

Policy information about studies involving animals; ARRIVE guidelines recommended for reporting animal research, and Sex and Gender in Research

Laboratory animals	For inflamed ear model, healthy female BALB/c mice (6-8 weeks old) were used. For DSS induced colitis, healthy female C57BL/6 mice (8-12 weeks old) were used. For biosafety analysis in vivo, male and female BALB/c mice (6-8 weeks old) were used.
Wild animals	No wild animal was used
Reporting on sex	Female mice were used in this study to ensure gender uniformity. The experiment was designed based on previous similar experiments (Chen, G. et al. Roe-inspired stem cell microcapsules for inflammatory bowel disease treatment. Proc. Natl Acad. Sci. USA 118 (2021); Wang, H. et al. Genetically engineered and enucleated human mesenchymal stromal cells for the targeted delivery of therapeutics to diseased tissue. Nat Biomed Eng 6, 882-897 (2022). It is expected that animal gender does not influence the results
Field-collected samples	No field-collected sample was used
Ethics oversight	The South China University of Technology Animal Care and Use Committee authorized all animal operations used in our research (2022020).

Note that full information on the approval of the study protocol must also be provided in the manuscript.

Flow Cytometry

Plots

Confirm that:

☒ The axis labels state the marker and fluorochrome used (e.g. CD4-FITC).

☒ The axis scales are clearly visible. Include numbers along axes only for bottom left plot of group (a 'group' is an analysis of identical markers).

☒ All plots are contour plots with outliers or pseudocolor plots.

☒ A numerical value for number of cells or percentage (with statistics) is provided.

Methodology

Sample preparation	Sample preparation listed in Methods.
Instrument	BD FACSCelesta
Software	Flowjo V10.0.7 was used for analysis.
Cell population abundance	No cell sorting was performed.
Gating strategy	For cell analysis, scatter-based gating is first performed on FSC-A/SSC-A to identify cells of interest based on size, followed by data gating on FSC-A/FSC-H to identify individual cells. Next, dead cells are excluded based on DAPI staining. In addition, Negative gating is confirmed by an antibody isotype control. T cells were gated according to the lineage marker CD3, then T cell types were gated in CD4, CD8, CD25, CD69 plots.

☐ Tick this box to confirm that a figure exemplifying the gating strategy is provided in the Supplementary Information.
